# H_2_O_2_ self-providing synergistic chemodynamic/photothermal therapy using graphene oxide supported zero valence iron nanoparticles†

**DOI:** 10.1039/d1ra04528h

**Published:** 2021-08-31

**Authors:** Miao Xu, Qin Li, Yi Xiang, Shanshan Yuan, Yihan Wu, Jing Zhang, Jinliang Liu, Xiaohui Zhu, Yong Zhang

**Affiliations:** School of Environmental and Chemical Engineering, Shanghai University Shanghai 200444 China xhzhu@shu.edu.cn biezy@nus.edu.sg; Department of Biomedical Engineering, Faculty of Engineering, National University of Singapore 117583 Singapore

## Abstract

Chemodynamic therapy (CDT) represents an emerging modality that treats cancer and other malignant diseases by using Fenton or Fenton-like catalysts to decompose hydrogen peroxide (H_2_O_2_) into toxic hydroxyl radicals (·OH). Despite its great promise, chemodynamic therapy is still limited by low endogenous H_2_O_2_ levels and lack of highly efficient nanocatalysts. In this study, we have developed multi-functional therapeutic nanocomposites GO–ZVI–GOx (GO = graphene oxide, ZVI = zero valence iron nanoparticles and GOx = glucose oxidase), where the GOx can catalyze the intracellular glucose and self-produce H_2_O_2_ for enhanced CDT therapy, and the GO is used as a template to avoid the aggregation of ZVI nanoparticles and also as an excellent photo-thermal converter for photothermal therapy under near-infrared (NIR) light. Our results show that this H_2_O_2_ self-generating nanoplatform can produce substantial amounts of reactive radicals under 808 nm NIR light due to the combinational effect of dual chemodynamic and photothermal therapy, which eventually leads to a significant decrease in cancer cell viability. It is believed that the methodology developed in this study enables conventional chemodynamic therapy to be efficiently improved, and holds great potential for overcoming challenges in many other H_2_O_2_-dependent cancer therapies.

## Abstract 2

A H_2_O_2_ self-providing therapeutic nanoplatform is reported to achieve enhanced chemodynamic/photothermal therapy.

## Graphical abstract

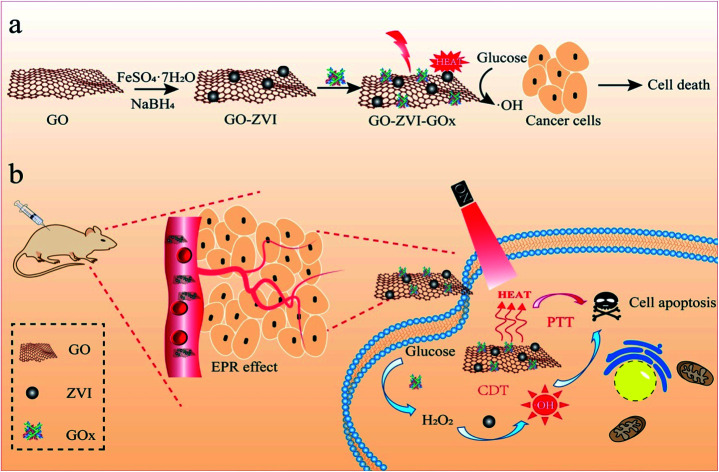

## 1. Introduction

Chemodynamic therapy (CDT) refers to the cancer treatment that utilizes chemical agents to catalyze endogenous hydrogen peroxide (H_2_O_2_) into highly reactive oxygen species (ROS) *via* the Fenton or Fenton-like reactions in order to induce cell death.^1–3^ Considering that the tumor microenvironment (TME) is characterized by mild acidity, Fenton-based CDT therapy is advantageous in its high tumor selectivity and specificity.^4,5^ Part of the reason is that the Fenton reaction is significantly limited by the slightly basic microenvironment in the normal tissue region.^6,7^ Therefore, CDT therapy has emerged as a promising strategy for selective intervention in cancer and other malignant diseases.^8,9^

To date, many types of metal ions (*e.g.*, Fe, Co, Ni, and Mn) have demonstrated their outstanding catalytic capability of decomposing H_2_O_2_ into toxic hydroxyl radicals (·OH).^1^ Excessive amounts of these reactive radicals can cause deleterious oxidative stress that can severely alter many types of bimolecular structures, such as cell membranes, proteins, lipids, deoxyribonucleic acid (DNA), *etc.*^10^ Among these Fenton reagents, zero-valence iron (ZVI) represents a class of iron-based nanoparticles which have been long applied in the degradation of organic pollutants,^11^ removal of heavy metal ions,^12^ and *in situ* remediation of soil,^13^ due to their exceptional properties including large surface-area-to-volume ratio, low standard potential (*E*_0_ = −0.44 V), and high reactivity.^14–16^

In addition, recent results have also shown promising prospective of ZVI for cancer therapy as an effective antitumor reagent. For example, Wu *et al.* reported that ZVI-based nanoparticles could selectively inhibit cancer cell but spare normal healthy ones,^17,18^ which was attributed to the cancer-specific cytotoxicity of the non-oxidized ZVI core in these nanoparticles.^19,20^ Later, amorphous ZVI nanoparticles were reported and used for cancer therapy by triggering the local Fenton reaction in the tumor region.^2^ In a more recent study, PVP (polyvinyl pyrrolidone) modified ZVI nanoparticles were demonstrated to have great potentials to serve as both the contrast agents for magnetic resonance imaging and the therapeutic reagents for synergetic cancer therapy.^21^ Despite the great progress, the application of ZVI-based Fenton therapy is still limited by the low endogenous H_2_O_2_ level, insufficient generation of ROS and easy aggregation of ZVI nanoparticles. Restricted by the low catalytic efficiency of Fenton reactions, it is still rather challenging to completely eliminate tumors relying solely on the ZVI-based CDT treatment. In this context, a synergistic therapy combining CDT and other treatment modality complimentary to CDT, would significantly amplify the therapeutic efficiency.

**Fig. 1:**
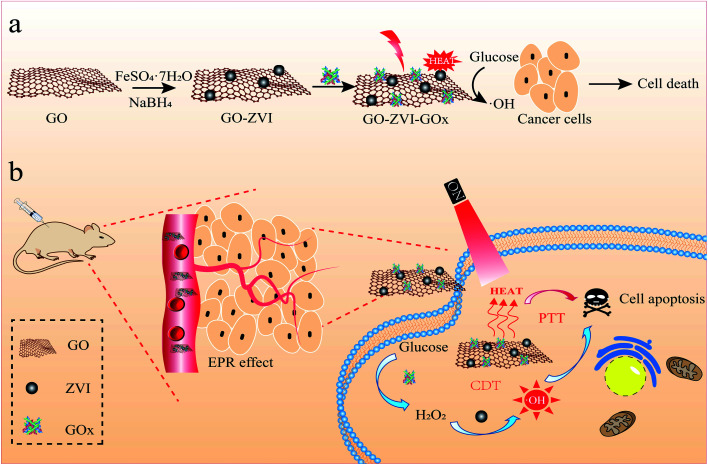
A schematic showing the synthesis of GO–ZVI–GOx nanocomposite (a) and its application in H_2_O_2_ self-providing chemodynamic/photothermal therapy (b).

Herein, we reported a self-supplying H_2_O_2_ nanoplatform that can simultaneously achieve both chemodynamic and photothermal (PTT) therapy. The specially designed nanoagent is composed of graphene oxide (GO) deposited by ZVI and GOx (glucose oxidase), where the GOx can catalyze the intracellular glucose into H_2_O_2_ and continuously provide H_2_O_2_ source for CDT treatment, thus removing the dependence of ZVI-based Fenton reaction on endogenous H_2_O_2_. Besides, GO is used as a template to prevent the ZVI from aggregating and also as a good photo-thermal converter for PTT therapy under near-infrared light (Fig. 1). The results show that substantial amount of ·OH radicals is generated upon excitation with the 808 nm laser light, as a result of the combinational effect of chemodynamic and photothermal therapy. Consequently, the growth of cancer cells is significantly prohibited. It is believed that the therapeutic nanoplatform developed in this work represents an efficient modality to resolve issues in the ZVI-based CDT treatment (*e.g.*, insufficient endogenous H_2_O_2_ level and easy aggregation of ZVI nanoparticles) and hold great potential for improved cancer therapy.

## 2. Experimental section

### 2.1 Materials

Potassium persulfate (K_2_S_2_O_8_) was purchased form Alfa and potassium permanganate (KMnO_4_) was purchased from Greagent. Phosphorus pentoxide (P_2_O_5_), ferrous sulfate heptahydrate (FeSO_4_·7H_2_O), ferric nitrate nonahydrate Fe(NO_3_)_3_·9H_2_O and 2,7-dichlorodi-hydrofluorescein diacetate (DCFH-DA) were purchased from Sigma-Aldrich. Graphite, sodium borohydride (NaBH_4_), 1-(3-dimethylaminopropyl)-3-ethylcarbodiimide hydrochloride (EDC), *N*-hydroxysuccinimide (NHS), potassium iodide (KI) and benzoic acid were purchased from Adamas. Glucose oxidase (GOx) was purchased from Aladdin. Cell culture medium (DMEM), fetal bovine serum (FBS) and penicillin-streptomycin were purchased from Gbico. Cell Counting Kit-8 (CCK-8) was purchased from Dojindo.

### 2.2 Synthesis of GO–ZVI

A modified Hummers method used to synthesize GO can be found elsewhere,^22,23^ while ZVI was prepared by a liquid-phase reduction method.^24,25^ In brief, the synthesis of GO–ZVI nanocomposite is as follows: 10 mL GO with a concentration of 5 mg mL^−1^ was added to 100 mL three-necked flask, and different amounts of FeSO_4_·7H_2_O was weighed and dissolved in the deionized water. Then, the dissolved FeSO_4_·7H_2_O was added to the above GO solution and stirred overnight to ensure the adsorption of Fe^2+^ ions on the GO surface. Next, the mixed solution was aerated with Ar gas for 30 minutes to remove the O_2_ from the flask, and then different amounts of NaBH_4_ were dropwise added into the mixed solution of GO and FeSO_4_·7H_2_O through the separator funnel. Then, the reaction was kept for another 30 minutes to ensure the sufficient production of ZVI nanoparticles on the GO surface. Next, the obtained black precipitate was centrifuged at 13 000 rpm for 15 minutes, and later dissolved in deoxygenated deionized water. The solution was again centrifuged at 4000 rpm for 5 minutes, the supernatant was retained. The process was repeated for 3 times. Finally, the samples of GO–ZVI with different GO/Fe ratios were sealed and stored. The procedure of preparation of ZVI was the same as that of GO–ZVI, but without the addition of GO.

### 2.3 Synthesis of GO–ZVI–GOx

The loading of GOx on GO–ZVI was performed by the assistance of EDC and NHS. Specifically, 10 μL EDC, 0.6 mg NHS and 0.2 mg GOx were first dissolved in PBS at a pH of 6.5, and constantly stirred for 2 hours. Then, 10 mL GO–ZVI was added to the above solution and stirred overnight. Next, the obtained GO–ZVI–GOx sample was centrifuged and washed three times with acid deoxygenated water and sealed for later use.

### 2.4 Synthesis of GO–Fe_2_O_3_

0.5 g Fe (NO_3_)_3_·9H_2_O was weighed and dissolved in 10 mL ultra-pure water. Then, GO solution with a concentration of 5 mg mL^−1^ was added dropwise to the above solution. After stirring, the solution was transferred to the drying oven at 60 °C for aging about 8 hours. Next, the products were washed with ultra-pure water for three times. After centrifugation at 13 000 rpm for 15 minutes, the precipitates were dried in the drying oven at 70 °C for later use.

### 2.5 *In vitro* detection of H_2_O_2_ produced by GO–ZVI–GOx

The generation of H_2_O_2_ was qualitatively detected by KI. This is because H_2_O_2_ can react with KI and produce I^3−^ ions, which has a strong absorption peak at around 350 nm. In brief, KI was first added to the aqueous solution with a glucose content of 20 μM, and then GO–ZVI–GOx was added to the above solution. After 10 minutes of reaction, the supernatant was centrifuged and its absorption intensity in the spectral region from 250 nm to 500 nm was measured.

### 2.6 *In vitro* detection of ·OH produced by GO–ZVI–GOx

Since the ·OH radicals produced by the Fenton reaction could react with benzoic acid (C_6_H_5_COOH) and form a fluorescent product, the benzoic acid was used as a probe to detect the generation of ·OH by GO–ZVI–GOx. Specifically, 100 μL glucose solution (2 mol mL^−1^) was added into the 10 mL benzoic acid solution. The pH of the mixture was adjusted to different values (*i.e.*, 7.5 and 6.4), followed by the addition of 500 μL GO–ZVI–GOx with the concentration of 2 mg mL^−1^. After 10 minutes, the mixture was centrifuged and the fluorescence of the supernatant was measured by the spectrofluorometer.

### 2.7 Evaluation of photothermal performance of GO–ZVI–GOx

The *in vitro* photothermal capability of as-prepared GO–ZVI–GOx under near infrared laser light was assessed as follows: 1 mL GO–ZVI–GOx solution with different concentrations (100, 200 and 400 μg mL^−1^) was placed 2.3 cm away from an 808 nm laser light and irradiated for six minutes at different power densities (1 W cm^−2^, 1.5 W cm^−2^, and W cm^−2^). The temperature of the solution upon irradiation was measure every 30 seconds using a thermocouple probe. The photothermal efficiency (*η*) was calculated by the following equations:^26^1
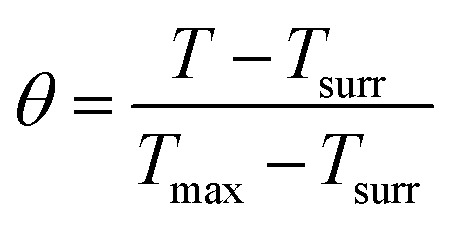

2 *t* = −*τ*_s_ ln *θ*

3
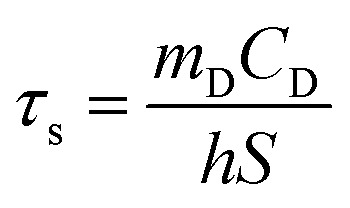
4
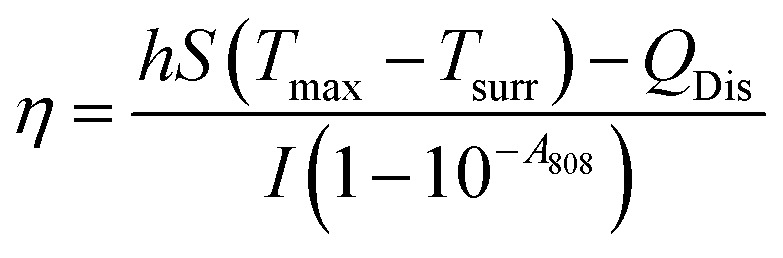
in which *T*_max_ is the maximum temperature (*i.e.*, 52.2 °C), *T*_surr_ is the surrounding temperature (*i.e.*, 23.3 °C). Then, the relation of *t* and –ln *θ* can be obtained according to eqn (2). The time constant of this system, *τ*_s_, is determined to be 207 s through the linear fitting. In eqn (3), *h* is heat transfer coefficient, *S* is the surface area of the cuvette, *m*_D_ and *C*_D_ are the mass and heat capacity of ultrapure water used as the solvent, which are 1 g and 4.2 J g^−1^. The *hS* in eqn (3) is 0.0203 mW per °C. In eqn (4), *Q*_Dis_ is the baseline energy induced by the sample cell, which is negligible because of its small value. *I* is the laser power (1.5 mW) and *A*_808_ is the absorption intensity of nanomaterials at 808 nm, which is measured to be 0.011. Finally, the photothermal efficiency (*η*) of GO–ZVI–GOx under 808 nm irradiation is 15.64% according to eqn (4).

### 2.8 Cytotoxicity of GO–ZVI–GOx

HeLa cell line was used to evaluate the toxicity of GO–ZVI–GOx. HeLa cells were cultured in DMEM medium containing 10% FBS, 100 units mL^−1^ of penicillin, and 100 μg mL^−1^ streptomycin and incubated in a 37 °C incubator with a moderate concentration of 95% and 5% carbon dioxide. The CCK-8 was used to detect the cell viability. HeLa cells were first seeded in 96-well plates with 8000 cells per well and the pH of cell culture medium DMEM was adjusted to be 7.4 and 6.5, respectively. After 24 hours of incubation, 100 μL no-glucose DMEM containing different concentrations of GO–ZVI–GOx (0, 12.5, 25, 50 100 and 200 μg mL^−1^) was added, and five parallel wells were prepared for each concentration. After continuous incubation for another 24 hours, the DMEM containing nanomaterials was discarded and the cells were rinsed with PBS for twice. Then, 100 μL DMEM containing 10% CCK-8 was added into each well to selectively stain the viable cell, and the cells were incubated for another 3 h. The absorption of each well at 450 nm was monitored by microplate reader.

### 2.9 Chemodynamic/photothermal therapy of GO–ZVI–GOx

HeLa cells were seeded in 96-well plates with 8000 cells per well and incubated for 24 hours. In order to assess the chemodynamic therapy alone, the high-glucose DMEM (pH = 7.4 and pH = 6.5) containing GO or GO–ZVI–GOx with different concentration (0, 12.5, 25, 50 100 and 200 μg mL^−1^) was added. Then, HeLa cells were incubated in 37 °C for another 24 hours and DMEM containing GO or GO–ZVI–GOx was replaced by fresh DMEM containing CCK-8. For the evaluation of synergistic chemodynamic and photothermal therapy, the culture medium was replaced with fresh DMEM (pH = 6.5) containing GO–ZVI–GOx with different concentration. Then 808 nm laser was irradiated for 5 minutes at a power density of 1.5 W cm^−2^. After incubation for 24 hours, the medium containing GO–ZVI–GOx was discarded and a fresh medium containing CCK-8 was added. The absorbance at 450 nm was tested by microplate reader.

### 2.10 Intracellular detection of ROS

A 2,7-dichlorodi-hydrofluorescein diacetate (DCFH-DA) probe was used to detect intracellular ROS generated by Fenton reaction because the DCFH-DA probe can be converted to DCF and emit strong green signals by the 488 nm light excitation. HeLa cells were first seeded in confocal dishes with 1.5 × 10^5^ per dish and incubated with PBS,GO or GO–ZVI–GOx for 24 hours. Then, 2 mL high-glucose DMEM containing 1 μL DCFH-DA solution was substituted for the previous DMEM and continued to be incubated for a total of 30 minutes. For the group of chemodynamic therapy, cells were washed with PBS for three times and then the fluorescence images under 488 nm light excitation were observed under confocal laser scanning microscope. For the group of dual chemodynamic/photothermal therapy, after washing cells with PBS for three times, the cells were irradiated with 808 nm at a power density of 1.5 W cm^−2^ for 5 minutes, and then the fluorescence imaging was observed under confocal laser scanning microscope.

### 2.11 Calcein-AM/PI staining

Calcein-AM (green fluorescence) and propidium iodide (PI, red fluorescence) was used to stain the live and dead HeLa cells, respectively. Briefly, HeLa cells were first seeded on 12 well plates at a density of 1.5 × 10^5^ and incubated with PBS,GO and GO–ZVI–GOx for 24 hours. Next, for the laser-treated group, cells were irradiated with 808 nm laser light (1.5 W cm^−2^) for 5 min. After incubation for another 4 hours, buffer solution containing Calcein-AM and PI was added and then the fluorescence imaging was observed under confocal laser scanning microscope.

### 2.12 Animal model and *in vivo* treatment

All animal procedures were performed in accordance with the guidelines for care and use of laboratory animals of Shanghai University and experimental protocols were approved by the Animal Ethics Committee of Shanghai University. Female BALB/c mice of 4 weeks were used for *in vivo* tumor therapy, which were purchased from Shanghai Laboratory Animals Center (SLAC, shanghai). The mouse tumor model was established by subcutaneously injecting 5 × 10^6^ HeLa cells into the right oxter of each mouse. When the tumor volume reached about 100 mm^3^, the *in vivo* therapy was conducted. Tumor-bearing mice were randomly divided into 6 groups, which were (1) PBS, (2) PBS + 808 nm light, (3) GO, (4) GO + 808 nm light, (5) GO–ZVI–GOx, (6) GO–ZVI–GOx + 808 nm light. Each group was injected with 100 μL of the corresponding samples every three days. For the groups of PBS + 808 nm light, GO + 808 nm light and GO–ZVI–GOx + 808 nm light, the 808 nm laser light (1.5 W cm^−2^) was applied for 5 minutes after each injection. Besides, body weight and tumor size were measured every two days. The normalized body weight was calculated as *m*/*m*_0_, where *m* and *m*_0_ represents the weight of each weighing and initial weight, respectively. The normalized tumor volume also calculated by the volume of each measuring to the initial volume, and the formula used to calculate the tumor volume is *V* = (*LW*^2^)/2, where *L* and *W* represents tumor length and width, respectively. After 14 days of treatment, the mice were sacrificed. Their vital organs, including heart, liver, spleen, lung, kidney and tumor, were removed and sectioned, stained with H&E, followed by histological analysis under microscope.

## 3. Results and discussions

### 3.1 Synthesis and characterization of GO–ZVI–GOx nanocomposites

**Fig. 2:**
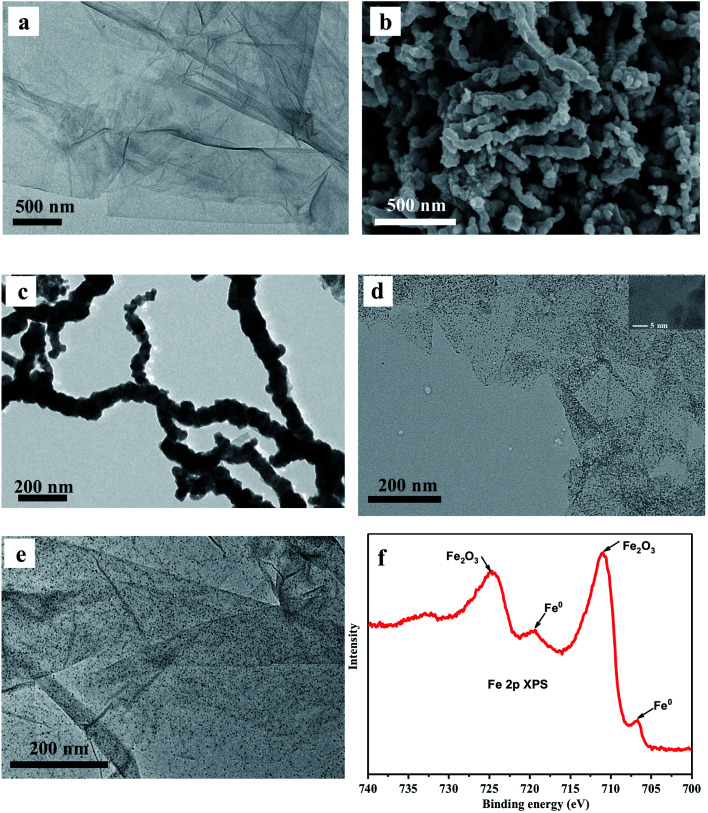
(a) TEM image of the graphene oxide (GO). (b and c) SEM and TEM image of as-synthesized zero valence iron (ZVI) nanoparticles. (d) TEM image of the GO–ZVI nanocomposite. Inset is the high magnified TEM image of GO–ZVI. (e) TEM image of the GO–ZVI–GOx nanocomposite. (f) XPS spectrum of the GO–ZVI–GOx nanocomposite.

The synthesis procedure of GO–ZVI–GOx nanocomposites mainly consists of three steps, as illustrated in Fig. 1a. Firstly, GO (graphene oxide) was obtained by a modified Hummers method,^22,23^ and then used as a template to grow ZVI (zero valence iron) nanoparticles and obtain the GO–ZVI nanostructures. Lastly, through EDC/NHS surface modification, the glucose oxidase enzyme (GOx) was conjugated with GO–ZVI due to the electrostatic forces. In Fig. 2a, the TEM (transmission electron microscope) image of GO shows that the as-prepared GO forms the shape of gauze with a large amount of wrinkles, suggesting that the GO exhibits a large specific surface area and can provide favorable nucleation sites for the growth of ZVI nanoparticles. Fig. 2b and c show the SEM (scanning electron microscopy) and TEM image of the as-synthesized ZVI nanoparticle, respectively, demonstrating that the ZVI nanoparticles are aggregated and particularly organized in the chain-like structure due to the magnetic dipole interactions.^27,28^ In order to solve the aggregation issue, the ZVI nanoparticles were *in situ* anchored on the GO surface, which process was mainly based on the redox reaction between the NaBH_4_ and Fe^2+^ ions adsorbed on GO surface. The corresponding chemical equation is as follow:Fe^2+^ + 2BH_4_^−^ + 6H_2_O → Fe^0^ + 2B(OH)_3_ + 7H_2_

Besides, the water solubility of the GO–ZVI nanocomposites should also be taken into consideration to meet the requirements for biological applications. Therefore, the optimum ratio of GO to ZVI is determined by comparing GO–ZVI nanocomposites with different Fe : GO ratios of 0.1 : 1, 0.5 : 1, 1 : 1 and 5 : 1. Fig. 2d presents the TEM image of the GO–ZVI sample with the Fe : GO ratio of 0.5 : 1 and Fig. S1† shows the TEM images of the GO–ZVI sample with the Fe : GO ratio of 0.1 : 1, 1 : 1, and 5 : 1. Clearly, with the increase of Fe : GO ratio, there is an increased amount of nanoparticles observed on the GO surface. For the GO–ZVI sample with the Fe : GO ratio of 0.5 : 1, the ZVI nanoparticles are uniformly distributed on the GO surface (Fig. 2d). Moreover, the higher magnification TEM image (inset in Fig. 2d) also confirms that the ZVI nanocrystals about 5–10 nm in size are deposited on the GO sheet. However, with the Fe : GO ratio higher than 0.5 : 1, the agglomeration of ZVI nanoparticles also becomes more pronounced, as shown in Fig. S1.† Water dispersity of the GO–ZVI nanocomposites are also compared among different Fe : GO ratios before and after 24 hour standing, see Fig. S2.† For the GO–ZVI samples with Fe : GO ratio of 0.1 : 1 and 0.5 : 1, no precipitation is observed at the bottom of the cuvette, while a large amount of precipitation appears for the GO–ZVI samples with Fe : GO ratio of 1 : 1 and 5 : 1. In view of the aggregation and water dispersity, the Fe : GO ratio of 0.5 : 1 is selected as the optimal value for preparing the GO–ZVI nanocomposites. The phase composition of the as-prepared GO–ZVI nanocomposites was further investigated by the X-ray diffraction (XRD) technique. In Fig. S3,† XRD patterns are compared for GO, ZVI and GO–ZVI samples, where GO–ZVI sample exhibits two major diffraction peaks at 2*θ* = 10.5° and 44.5°, corresponding to the (001) crystal plane of GO and (110) crystal plane of ZVI nanoparticles. This is another confirmation of successful deposition of the ZVI nanoparticles onto the GO surface. Following the synthesis of GO–ZVI, the EDC/NHS modified GOx is further anchored onto the GO-ZVI nanocomposites. Fig. 2e presents the TEM image of the obtained GO–ZVI–GOx nanocompounds with ZVI nanoparticles still remain uniformly distributed throughout the GO surface, suggesting that the GOx decoration does not interrupt the distribution of ZVI nanoparticles in the system. In Fig. 2f, X-ray photoelectron spectra (XPS) of the GO–ZVI–GOx nanocomposites are obtained in which the peaks at around 706.9 and 720.2 eV correspond to the Fe^0^ and peaks at 711.1 and 725.1 eV indicate the existence of Fe_2_O_3_. Since XPS technique is very sensitive to the surface structures, these results suggest that the surface of ZVI nanoparticles are slightly oxidized to iron oxide.^15^ Notably, previous studies have also reported that the non-oxidized ZVI core still exhibits the cancer-specific cytotoxicity regardless of surface oxidization.^19,20^

**Fig. 3:**
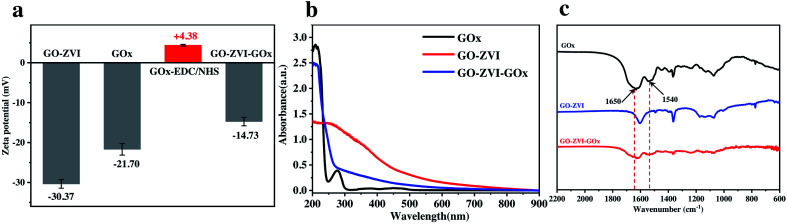
(a) Comparison of zeta potential of GO–ZVI, GO, GOx–EDC/NHS, and GO–ZVI–GOx. (b) UV-vis spectra of GOx, GO–ZVI and GO–ZVI–GOx nanocomposites. (c) IR spectra of GOx, GO–ZVI and GO–ZVI–GOx. The two black arrows indicate the amide I and amide II band of the amide group of GOx compound.

In order to verify the successful loading of GOx on the GO–ZVI, the zeta potential and UV-vis absorption spectra of GO–ZVI–GOx were then recorded. As shown in Fig. 3a, the zeta potential of GO–ZVI and GOx is measured to be −30.37 mV and −21.7 mV, respectively. However, after surface modification by EDC/NHS, the zeta potential of GOx-EDC/NHS changes to be +4.38 mV due to the introduction of positively charged amine group. The reason why GOx was first modified with EDC/NHS was to increase the stability and conjugation yield.^29^ In addition, the electrostatic attractions between the oppositely charged surface of GO–ZVI and GOx–EDC/NHS would lead to the formation of GO–ZVI–GOx nanocomposite upon mixing of the two solutions.

Fig. 3b shows the UV-vis absorption spectra of GOx, GO–ZVI and GO–ZVI–GOx, which clearly demonstrates the GO–ZVI–GOx and pure GOx samples have similar features in the spectral region from 200 nm to 300 nm. However, the GOx-free GO–ZVI sample exhibits no evident absorption signals within this range. More importantly, the infrared spectra in Fig. 3c also indicates that both GO–ZVI–GOx and GOx display evident features around 1650 cm^−1^ and 1540 cm^−1^ (indicated by the black arrows) corresponding to the amide I and amide II band of the amide group of GOx, respectively.^30^ Besides, we have also studied the stability of GO–ZVI–GOx in different solutions before and after 24 hour standing (Fig. S4†). As shown in Fig. S4,† no precipitates are observed for the four solutions (*i.e.*, H_2_O, PBS, ethanol, and DMEM) after standing for 24 hours, indicating the good stability of as-prepared GO–ZVI–GOx nanocomposites. Particularly, the good solubility and stability of GO–ZVI–GOx nanocomposites in DMEM also allows for the subsequent *in vitro* experiments.

### 3.2 Evaluation of H_2_O_2_ productivity by GO–ZVI–GOx nanocomposites

**Fig. 4:**
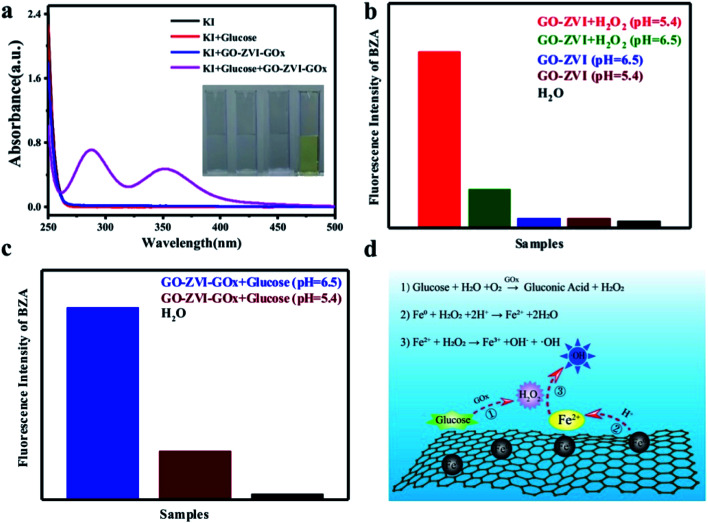
(a) Comparison of UV-vis spectra for different groups: KI solution, KI + glucose, KI + GO–ZVI–GOx, and KI + glucose + GO–ZVI–GOx. (b) Integrated fluorescence intensity of benzoic acid (BZA) solution containing H_2_O and GO–ZVI in the presence or absence of H_2_O_2_ at different pH values (*i.e.*, 5.4 and 6.5); (c) Integrated fluorescence intensity of benzoic acid (BZA) solution containing GO–ZVI–GOx and glucose at different pH values (*i.e.*, 5.4 and 6.5). (d) Proposed mechanism of Fenton reaction based on GO–ZVI–GOx nanocomposites.

The capability of the as-prepared GO–ZVI–GOx to produce H_2_O_2_ was evaluated using the KI reagent. With the existence of H_2_O_2_, the KI can be reduced to I^3−^, which has a prominent absorption around 270–400 nm and can thus be used as a probe to detect H_2_O_2_ generation.^31^ As shown in Fig. 4a, there is no evident absorption in the spectral range from 250 nm to 400 nm for the sample groups of KI (black), KI + glucose (red), and KI + GO–ZVI–GOx (blue). However, for the group of KI + glucose + GO–ZVI–GOx (purple), obvious absorption peaks appeared around 290 nm and 350 nm, suggesting the generation of H_2_O_2_. In addition, only the solution treated with the group of KI + glucose + GO–ZVI–GOx turns to light-yellow among all the four tested samples (inset in Fig. 4a). This is a clear indication that the capability of GOx to catalyze glucose to H_2_O_2_ is still remained after being loaded onto the GO–ZVI surface.

### 3.3 Investigation of Fenton activity by GO–ZVI–GOx nanocomposites

The Fenton activity of the GO–ZVI–GOx nanocomposites to generate reactive radicals (*e.g.*, ·OH) was further evaluated by the benzoic acid. Notably, the benzoic acid (BZA) can be readily oxidized by the reactive ·OH radicals and produce hydroxybenzoic acid characterized with high fluorescence in the spectral range from 350 nm to 500 nm.^32^ As a result, the generation of reactive radicals during the Fenton reaction can be effectively monitored by measuring the variations of fluorescence intensity of benzoic acid.

Fig. 4b compares the integrated fluorescence intensity of benzoic acid solution containing GO–ZVI in the presence or absence of H_2_O_2_ at different pH values (*i.e.*, 5.4 and 6.5). It can be seen that there are no significant fluorescence signals without the existence of H_2_O_2_, independent of the pH of the solutions. In contrast, an evident increase in the fluorescence is observed in the presence of H_2_O_2_. Particularly, the fluorescence intensity at pH of 5.4 is much stronger than that at pH of 6.5, suggesting the pH-dependent performance of Fenton reactions.^33,34^Fig. 4c compares the integrated fluorescence intensity of benzoic acid solution containing GO–ZVI–GOx and glucose solution at pH 5.4 (dark red) and pH 6.5 (blue), both of which show adequate fluorescence signals even without the presence of H_2_O_2_, indicating good capability of GOx to catalyze glucose and produce H_2_O_2_ for Fenton reactions. Besides, with lower pH, much higher fluorescence intensity is observed. This pH-related Fenton behavior is crucial for the efficient and precise chemodynamic therapy, as the microenvironments of the cancer cells are characterized with lower pH than normal cells. Based on these observations, the proposed pathway of Fenton reactions for GO–ZVI–GOx nanocomposite is as follows (Fig. 4d):

(1) Glucose in the solution is firstly catalyzed to H_2_O_2_ by GOx anchored on the GO–ZVI surface.

(2) The highly active ZVI nanoparticles are oxidized to Fe^2+^ ions by the H_2_O_2_ in the acidic environment.

(3) The newly formed Fe^2+^ ions further react with H_2_O_2_ and ultimately generate a large amount of ·OH radicals.

### 3.4 Photothermal evaluation by GO–ZVI–GOx nanocomposites under NIR light

**Fig. 5:**
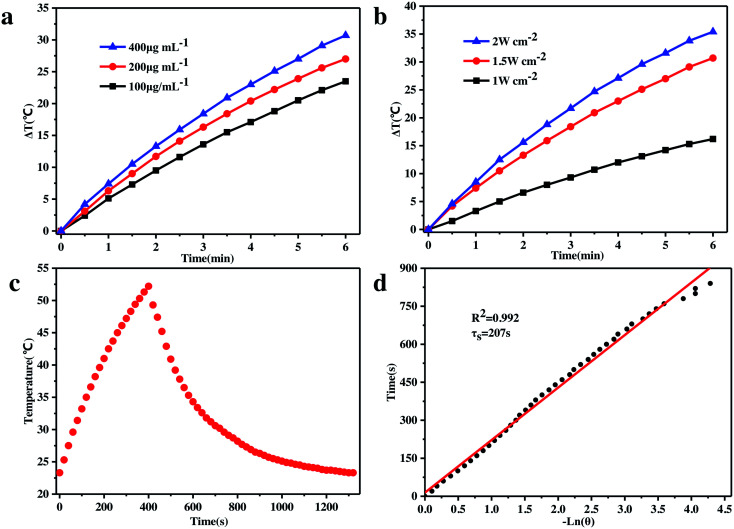
(a) Temperature variations of GO–ZVI–GOx solution with different concentrations (100 μg mL^−1^, 200 μg mL^−1^, and 400 μg mL^−1^) illuminated by 808 nm light at a power density of 2.0 W cm^−2^. (b) Temperature changes of the GO–ZVI–GOx solution (200 μg mL^−1^) upon 808 nm laser light at different power densities (1 W cm^−2^, 1.5 W cm^−2^ and 2 W cm^−2^). (c) Temperature variations of the GO–ZVI–GOx solution upon 808 nm laser light irradiation for 400 seconds, followed with the cooling process by turning off the laser light. (d) Fitting of the time constant corresponding to the heat transfer from the system.

It has long been recognized that the two major components, *i.e.*, GO and ZVI, of the as-prepared GO–ZVI–GOx nanocomposite are promising nanoagents for photothermal therapy, owing to their high absorbance at the NIR window, outstanding photo-thermal conversion efficiency and strong photostability. Therefore, the *in vitro* photothermal capability of our GO–ZVI–GOx composite was evaluated through exposure to the 808 nm laser light. As shown in Fig. 5a, the temperature of the test solution is positively related to the concentration of GO–ZVI–GOx nanocomposites. For example, the temperature was elevated by 22 °C in six minutes when the concentration of GO–ZVI–GOx was 100 μg mL^−1^, and by 27 °C when the concentration of GO–ZVI–GOx was 200 μg mL^−1^. Fig. 5b presents the temperature variation *via* irradiation by 808 nm laser light at different power densities (1.0 W cm^−2^, 1.5 W cm^−2^, and 2.0 W cm^−2^) with the same concentration of GO–ZVI–GOx (200 μg mL^−1^). As expected, the temperature rises with an increase in the laser power density. The maximum temperature increase reaches around 35 °C after exposure to the 808 nm laser light for six minutes at the power density of 2.0 W cm^−2^. In order to further evaluate the photothermal conversion efficiency of GO–ZVI–GOx, the GO–ZVI–GOx solution was exposed to the 808 nm laser light for 400 seconds. The temperature change of the GO–ZVI–GOx solution was then recorded while the laser light was off (Fig. 5c). The fitted time constant for the heat transfer from the system is determined to be 207 s and the photothermal conversion efficiency(η) is calculated to be 15.64% (Fig. 5d), which is comparable to some conventional photothermal agents such as semiconducting polymer nanoparticles (15.8%)^35^ and higher than gold nanoshells (13%).^36^ This high photothermal conversion efficiency under 808 nm NIR light is believed to endow the as-prepared GO–ZVI–GOx nanocomposites with good potential for PTT therapy of tumor cells. Notably, the *η* for the GO–ZVI sample is calculated to be 16.62% (Fig. S5†), also indicating that the GOx loading on GO–ZVI has neglectable effect on the photothermal conversion efficiency.

### 3.5 Comparison of GO–ZVI and GO–Fe_2_O_3_

**Fig. 6:**
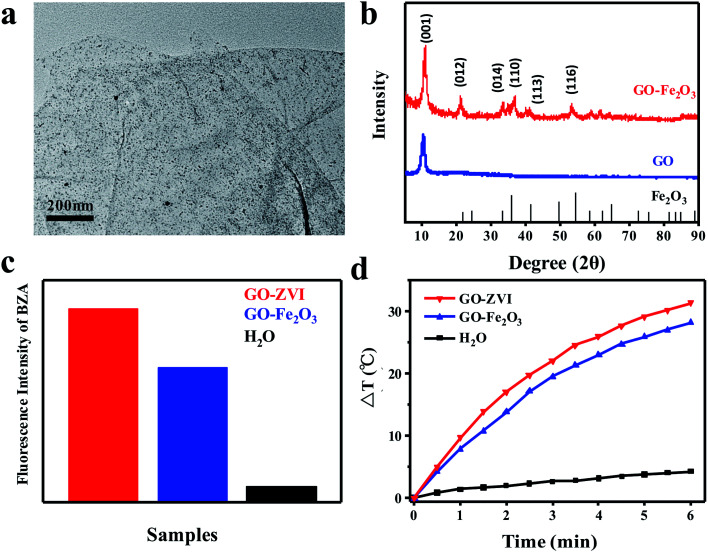
(a) TEM image of GO–Fe_2_O_3_ nanocomposites. (b) XRD patterns of GO–Fe_2_O_3_ in a comparison with GO and Fe_2_O_3_. (c) Integrated fluorescence intensity of benzoic acid solution containing GO–ZVI and GO–Fe_2_O_3_ in the presence of H_2_O_2_. (d) Temperature variations of GO–ZVI (200 μg mL^−1^) and GO–Fe_2_O_3_ solution (200 μg mL^−1^) and H_2_O illuminated by 808 nm light at a power density of 2.0 W cm^−2^.

In addition, we have also synthesized GO–Fe_2_O_3_ nanocomposites for a better comparison with GO–ZVI in the aspects of Fenton activity and photothermal capabilities. Fig. 6a presents the TEM image of as-prepared GO–Fe_2_O_3_ nanocomposites, which demonstrates that the Fe_2_O_3_ nanoparticles are uniformly distributed on the GO surface. In Fig. 6b, the XRD patterns of GO–Fe_2_O_3_ are compared with GO and Fe_2_O_3_, where the GO– Fe_2_O_3_sample exhibits several diffraction peaks that correspond to different crystallographic planes of GO and Fe_2_O_3_. This confirms the successful deposition of the Fe_2_O_3_ nanoparticles onto the GO surface. Next, the Fenton catalytic activity of GO–ZVI and GO–Fe_2_O_3_ is compared. Similar to Fig. 4, benzoic acid (BZA) is also used to evaluate the ·OH radicals generated during the Fenton reaction. Fig. 6c compares the integrated fluorescence intensity of benzoic acid solution containing GO–ZVI, GO–Fe_2_O_3_ and H_2_O in the presence of H_2_O_2_. Clearly, it can be obtained that the fluorescence intensity of GO–ZVI is about one third time stronger than that of GO–Fe_2_O_3_. This is because the oxidization of ZVI can produce large amount of Fe^2+^, which promotes more generation of hydroxyl radicals^37^*.*Fig. 6d presents the temperature variations of GO-ZVI solution (200 μg mL^−1^), GO-Fe_2_O_3_ solution (200 μg mL^−1^) and H_2_O illuminated by 808 nm light (2W cm^−2^), which also demonstrates that the temperature rises higher in the GO-ZVI solution than the GO–Fe_2_O_3_ solution. Therefore, it can be obtained that the GO-ZVI nanocomposite exhibits better performance than GO–Fe_2_O_3_ in the aspects of both Fenton activity and photothermal capability.

### 3.6 *In vitro* synergistic chemodynamic/photothermal therapy by GO–ZVI–GOx

**Fig. 7:**
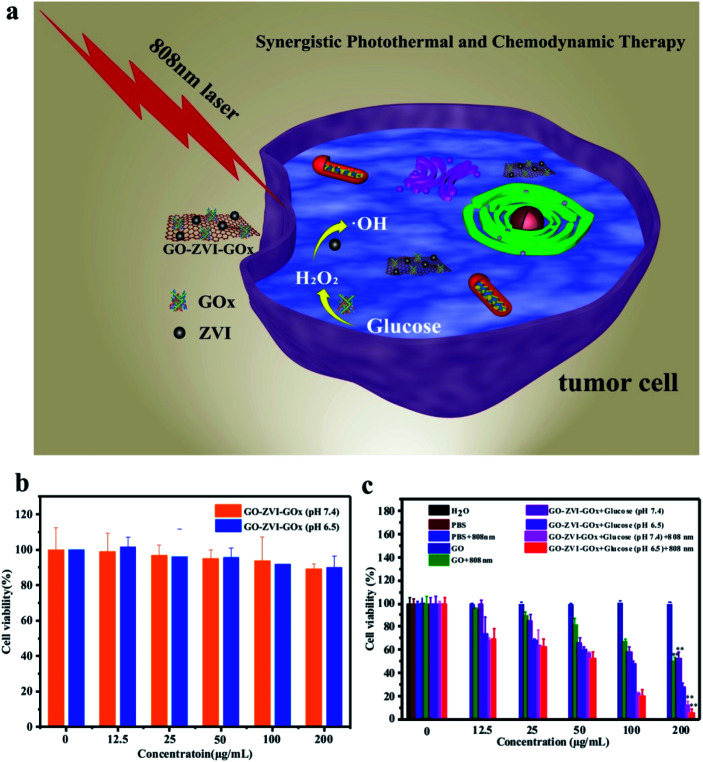
(a) Schematic cartoon of the synergistic chemodynamic/photothermal therapy by GO–ZVI–GOx nanocomposites. (b) Cell viability of GO–ZVI–GOx nanocomposites with different concentrations at pH 7.4 (yellow) and 6.5 (blue). (c) Cell viability of HeLa cells incubated with GO–ZVI–GOx nanocomposites with different concentrations for 24 hours (**p < 0.01). The incubated cells were divided into the following groups: PBS control group, PBS + 808 nm light, GO + glucose, GO + glucose + 808 nm light, GO–ZVI–GOx + glucose (pH 7.4), GO–ZVI–GOx + glucose (pH 6.5), GO–ZVI–GOx + glucose (pH 7.4) + 808 nm light, and GO–ZVI–GOx + glucose (pH 6.5) + 808 nm light.

The *in vitro* synergistic chemodynamic/photothermal therapy of GO–ZVI–GOx nanocomposites is assessed using the HeLa human cell line, as illustrated in Fig. 7a.

On one hand, Fenton-based chemodynamic therapy is initiated by decomposing the H_2_O_2_ into ·OH radicals once the GO–ZVI–GOx enters the mildly acidic microenvironment of the cancer cells. The H_2_O_2_ can also be additionally provided by GO–ZVI–GOx nanocomposites *via* the catalysis of glucose into H_2_O_2_. On the other hand, upon the illumination by the 808 NIR laser light, the high photo-thermal conversion efficiency of GO–ZVI–GOx enables the local hyperpyrexia and further increases the amount of cytotoxic radicals. Notably, it is of significance to evaluate the cytotoxicity of GO–ZVI–GOx nanocomposites prior to performing the *in vitro* treatment. In this study, the standard CCK-8 assay was conducted to assess cell viabilities. Fig. 7b presents the viability of HeLa cells co-incubated with different concentrations of GO–ZVI–GOx (12.5, 25, 50 100 and 200 μg mL^−1^) at two pH values (7.4 and 6.5). Apparently, the cytotoxicity of GO–ZVI–GOx is rather low at concentrations below 100 μg mL^−1^, regardless of pH values. Over 90% cell viability was achieved with GO–ZVI–GOx concentration up to 200 μg mL^−1^, indicating low cytotoxicity of the GO–ZVI–GOx to HeLa cells even at high doses. Following the cytotoxicity test, the chemodynamic/photothermal therapeutic effect of GO–ZVI–GOx in the presence of glucose is evaluated at the cellular level. Fig. 7c shows the viability results of HeLa cells incubated with GO and GO–ZVI–GOx under different treatments. As shown in Fig. 7c, no evident cell damages are observed in the control groups including, H_2_O, PBS, PBS + 808 nm and GO alone without light illumination. However, when the HeLa cells are treated with GO solution (200 μg mL^−1^) and 808 nm light irradiation, the cell viability is reduced to 50%, indicating the photothermal (PTT) effect of GO under 808 nm light irradiation. Similarly, when the HeLa cells are incubated with GO–ZVI–GOx (200 μg mL^−1^) in the presence of glucose, the cell viability is about 50% at pH 7.4 and 30% at pH 6.5, which explicitly shows the chemodynamic (CDT) effect of GO–ZVI–GOx. In a comparison with the cell viability in Fig. 7b, it indicates the important roles that acid condition and glucose play in the Fenton reaction by GO–ZVI–GOx. Since Fenton reaction is pH dependent, lower pH promotes the reaction and thus generates more cytotoxic · radicals. Meanwhile, the added glucose can be catalyzed by GO–ZVI–GOx into H_2_O_2_ which is one of the important prerequisites for the Fenton reaction. The additional exposure of 808 nm laser light leads to further drop of the cell viability to below 5%, in the presence of GO–ZVI–GOx (200 μg mL^−1^), demonstrating its high inhibition efficiency for the HeLa cells. This can be explained by the fact that the laser exposure increases the temperature and further boosts the generation of ·OH radicals,^21^ leading to the substantial decrease in the cell viability.

**Fig. 8:**
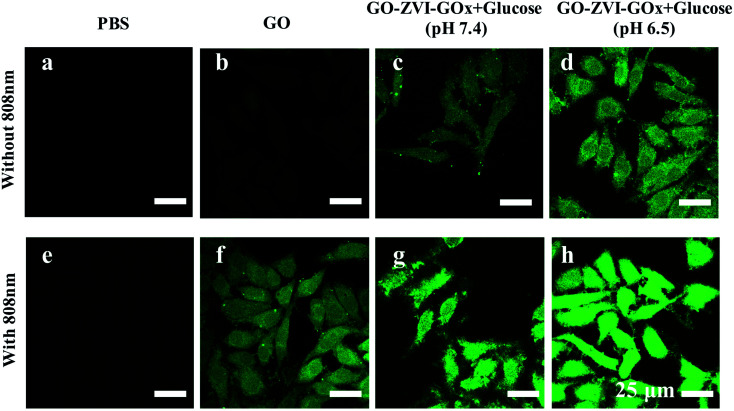
Confocal imaging of HeLa cells stained by DCFH-DA probes after different treatments, including PBS, PBS + 808 nm, GO, GO + 808 nm, GO–ZVI–GOx + glucose (pH 7.4), GO–ZVI–GOx + glucose (pH 6.5), GO–ZVI–GOx + glucose (pH 7.4) + 808 nm light, and GO–ZVI–GOx + glucose (pH 6.5) + 808 nm light.

In order to visualize the *in vitro* production of ROS signals by the GO–ZVI–GOx, the 2′,7′-dichlorfluorescein-diacetate (DCFH-DA) probe was used to detect the intracellular ROS as it could be oxidized to DCF and released the strong green emissions upon 488 nm excitation.^38,39^ As shown in Fig. 8, for the group treated with PBS, PBS + 808 nm and GO, the green fluorescence signals is rather weak. However, for HeLa cells treated with GO and 808 nm light illumination (GO + 808 nm), the green fluorescence is identifiable, which is mainly attributed to the good photothermal effect of GO. For the group treated with GO–ZVI–GOx nanocomposites in the presence of glucose at pH of 7.4, adequate fluorescence signals are observed, while more ROS signals can be identified at a lower pH of 6.5. Besides, much stronger fluorescence signals are generated when the cells are additionally exposed to irradiation with the 808 nm light. Particularly, the strongest fluorescence signals can be observed for the cells treated with GO–ZVI–GOx nanocomposites under 808 nm light illuminations at pH of 6.5 (*i.e.*, GO–ZVI–GOx + glucose (pH 6.5) + 808 nm light). These results are consistent with the significant decrease in the cell viability under light exposure (Fig. 7c), confirming the increased generation of ROS through the synergistic effect of dual chemodynamic and photothermal therapy based on GO–ZVI–GOx nanocomposites.

**Fig. 9:**
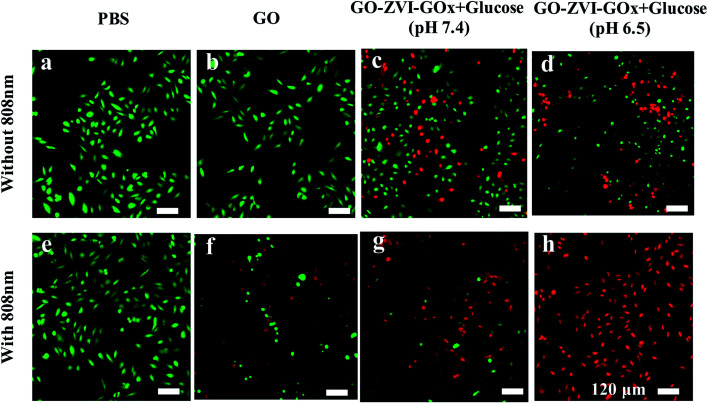
Confocal imaging of living and dead cells stained by Calcein-AM/PI probes after different treatments, including PBS, PBS + 808 nm, GO, GO + 808 nm, GO–ZVI–GOx + glucose (pH 7.4), GO–ZVI–GOx + glucose (pH 6.5), GO–ZVI–GOx + glucose (pH 7.4) + 808 nm light, and GO–ZVI–GOx + glucose (pH 6.5) + 808 nm light.

Additionally, we have also used Calcein-AM (green fluorescence) and propidium iodide (PI, red fluorescence) to stain the live and dead HeLa cells, respectively. As shown in Fig. 9, in the absence of GO, the 808 nm light irradiation does not cause any evident damage to cells. Similarly, HeLa cells only treated with GO solution (200 μg mL^−1^) exhibit strong green fluorescence, indicating the GO has no negative influence on cell viability. However, when the HeLa cells are treated with GO solution (200 μg mL^−1^) and 808 nm light irradiation, red fluorescence signals (dead cells) appear, which is due to the photothermal effect of GO under 808 nm light irradiation. Besides, for the HeLa cells only exposed to GO–ZVI–GOx solution (200 μg mL^−1^), dead cells can already be observed. Particularly, higher number of HeLa cells are killed at pH 6.5 than 7.4, suggesting that more hydroxyl radicals are produced in the more acidic environment. These results are also consistent with the *in vitro* results of CCK-8 (Fig. 7c) and ROS detection (Fig. 8). Most importantly, when the cells were treated with GO–ZVI–GOx solution (200 μg mL^−1^) and 808 nm light irradiation, nearly all the cells exhibited strong red fluorescence, indicating the synergistic photothermal/chemodynamic therapy of as-prepared GO–ZVI–GOx nanocomposites at 808 nm light irradiations.

### 3.7 *In vivo* treatment

**Fig. 10:**
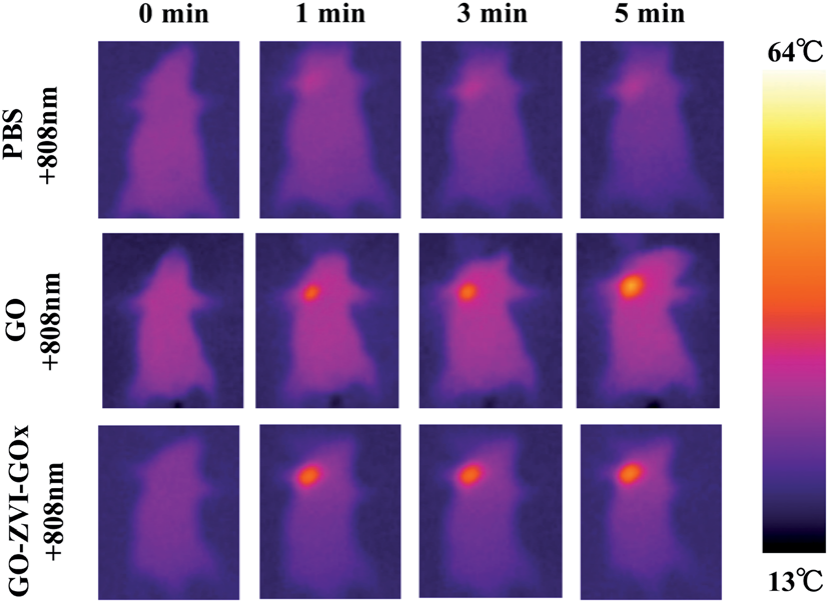
Infrared thermal images of mice injected with PBS, GO and GO–ZVI–GOx solution under different irradiation time of 808 nm laser light.

The small mouse model was established by intratumorally injecting HeLa cells into mice to verify the therapeutic effect of GO–ZVI–GOx *in vivo*. Firstly, in order to investigate the photothermal effect of the as-prepared materials *in vivo*, the temperature change of tumor sites in three groups of tumor-bearing mice was recorded by infrared thermal imager under different irradiation time of 808 nm laser (1.5 W cm^−2^, 5 minutes). As shown in Fig. 10, when only PBS solution was injected into the tumor, the temperature of the tumor site did not significantly increase. However, when the solution of GO or GO–ZVI–GOx sample was injected, the temperature of the tumor site evidently increased over irradiation time. Specifically, after 5 minutes of irradiation under 808 nm light, the temperature could reach over 50 °C and 45 °C, respectively. Suggesting a good photothermal conversion capability of as-prepared GO–ZVI–GOx samples.

**Fig. 11:**
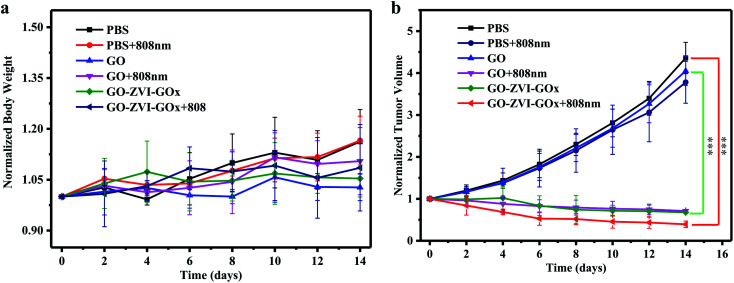
The trend of changes in body weight (a) and tumor volume (b) for treated mice over the 14 day treatment (*n* = 5, mean ± s.d., ***P < 0.001).

Next, the therapeutic effect of GO–ZVI–GOx samples are systematically investigated. When the tumor reached about 100 mm^3^, tumor-bearing mice were randomly divided into six groups,: (1) only injection of PBS, (2) injection of PBS and irradiated with 808 nm laser light, (3) only injection of GO, (4) injection of GO and irradiated with 808 nm laser light, (5) only injection of GO–ZVI–GOx, (6) injection of GO–ZVI–GOx and irradiated with 808 nm laser light. Notably, it would be be ideal to detect the production of ·OH radicals during the treatment, which, however, still requires reliable and artefact-free methods to quantify the amount of ·OH radicals *in vivo*.^40^ Therefore, only the body weight and tumor volume of the mice were measured every two days. Fig. 11a and Fig. 11b shows the trend of changes in body weight and tumor volume, respectively. It can be obtained that the body weight of the tumor-bearing mice in all groups increased slightly compared with the initial body weight, indicating that the injection of GO and GO–ZVI–GOx had neglectable effects on the health of the tumor-bearing mice. However, regarding the change of tumor volume, for the group treated with PBS alone (PBS), the tumor size was about 4.5 times larger than the initial value. Similarly, for the group treated with PBS and 808 nm irradiation (PBS + 808 nm), the tumor size also grew rapidly, indicating that 808 nm irradiation alone did not have effective prohibition ability on tumor growth. For the mice treated with GO + 808 nm light and GO–ZVI–GOx, the size of tumor after treatment was slightly reduced. However, for the group treated with GO–ZVI–GOx under 808 nm light irradiation, the tumor size was significantly smaller and the tumor size was about only half of the initial size after 14 days of treatment. In addition, Fig. S6† shows the photographs of the excised tumors from mice, which also indicates that the treatment with GO–ZVI–GOx under 808 nm irradiation substantially prohibited the tumor growth with the smallest tumor size. All these results suggest that GO–ZVI–GOx can effectively inhibit the tumor growth under 808 nm light, due to the synergistic therapeutic effects of chemodynamic and photothermal therapy.

**Fig. 12:**
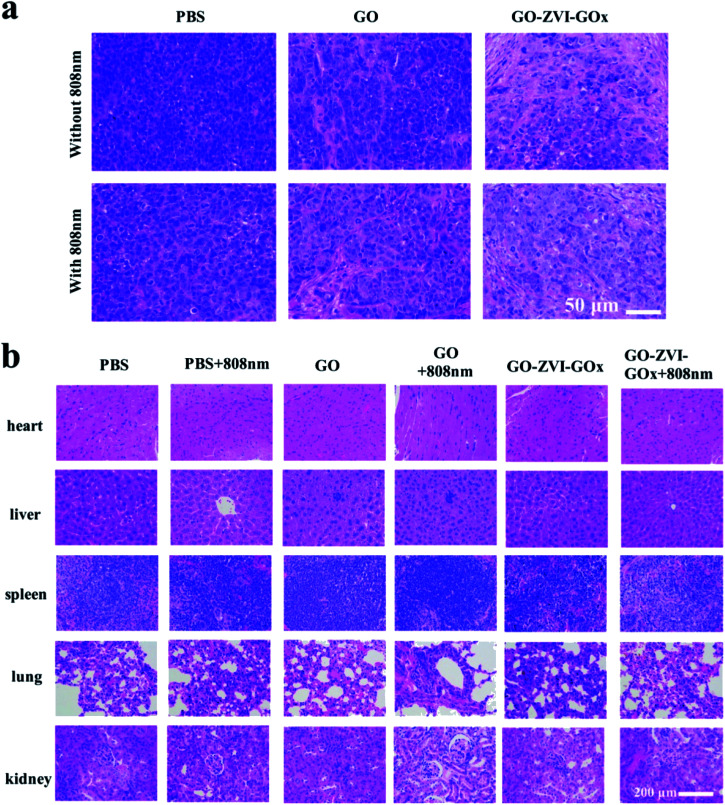
H&E staining of tumor (a) and vital organs (heart, liver, spleen, lung, and kidney) (b) of mice after 14 days of treatment.

In order to further confirm the therapeutic effect, histological analysis was carried out on tumor tissus (Fig. 12a). Compared with control groups (*i.e.*, PBS, PBS + 808 nm, GO), the groups with treatment (*i.e.*, GO + 808 nm, GO–ZVI–GOx and GO–ZVI–GOx + 808 nm) showed evident tumor tissue damages. Particularly, the group treated with GO–ZVI–GOx + 808 nm showed most severe apoptosis and cell shrinking. Fig. 12b presents the H&E staining of several major organs (heart, liver, spleen, lung, and kidney), which all maintain normal physiological states without obvious damages, indicating that GO–ZVI–GOx has neglectable short-term toxicity in mice.

## 4. Conclusions

In this study, a novel H_2_O_2_ self-providing dual chemodynamic/photothermal therapeutic nanoplatform was reported, in which zero valence iron nanoparticles (ZVI) and glucose oxidase (GOx) were deposited on graphene oxide (GO). On one hand, GO functions as a template to anchor ZVI nanoparticles for chemodynamic therapy and GOx for self-generating H_2_O_2_*via* the catalysis of glucose to further enhance chemodynamic therapy. On the other hand, GO and ZVI could also act as efficient photothermal conversion agents for photothermal therapy under 808 nm laser light. Due to such synergistic effect of chemodynamic and photothermal therapies, a substantial amount of ·OH signals can be generated upon 808 nm laser light irradiation, which eventually resulted in the significant decrease in viability of cancer cells. It is believed that the strategically designed synergistic nanoplatform based on ZVI and GO could be potentially utilized for cancer therapy in the future.

## Conflicts of interest

The authors declare no competing financial interest.

## Supplementary Material

## References

[cit1] Wang X., Zhong X., Liu Z., Cheng L. (2020). Recent progress of chemodynamic therapy-induced combination cancer therapy. Nano Today.

[cit2] Zhang C., Bu W., Ni D., Zhang S., Li Q., Yao Z., Zhang J., Yao H., Wang Z., Shi J. (2016). Synthesis of iron nanometallic glasses and their application in cancer therapy by a localized Fenton reaction. Angew. Chem., Int. Ed..

[cit3] Lin L.-S., Song J., Song L., Ke K., Liu Y., Zhou Z., Shen Z., Li J., Yang Z., Tang W., Niu G., Yang H.-H., Chen X. (2018). Simultaneous Fenton-like ion delivery and glutathione depletion by MnO_2_-based nanoagent to enhance chemodynamic therapy. Angew. Chem., Int. Ed..

[cit4] Wang L., Huo M., Chen Y., Shi J. (2018). Tumor Microenvironment-enabled nanotherapy. Adv. Healthcare Mater..

[cit5] Zhu S., Gu Z., Zhao Y. (2018). Harnessing tumor microenvironment for nanoparticle-mediated radiotherapy. Adv. Ther..

[cit6] Murphy M. P. (2009). How mitochondria produce reactive oxygen species. Biochem. J..

[cit7] Tang Z., Liu Y., He M., Bu W. (2019). Chemodynamic therapy: tumour microenvironment-mediated Fenton and Fenton-like reactions. Angew. Chem., Int. Ed..

[cit8] Xuan W., Xia Y., Li T., Wang L., Liu Y., Tan W. (2020). Molecular self-assembly of bioorthogonal aptamer-prodrug conjugate micelles for hydrogen peroxide and pH-independent cancer chemodynamic therapy. J. Am. Chem. Soc..

[cit9] Hu Y., Lv T., Ma Y., Xu J., Zhang Y., Hou Y., Huang Z., Ding Y. (2019). Nanoscale coordination polymers for synergistic NO and chemodynamic therapy of liver cancer. Nano Lett..

[cit10] Pham-Huy L. A., He H., Pham-Huy C. P. (2008). Free radicals, antioxidants in disease and health. Int. J. Biomed. Sci..

[cit11] Xie P., Guo Y., Chen Y., Wang Z., Shang R., Wang S., Ding J., Wan Y., Jiang W., Ma J. (2017). Application of a novel advanced oxidation process using sulfite and zero-valent iron in treatment of organic pollutants. Chem. Eng. J..

[cit12] Li S., Yang F., Li J., Cheng K. (2020). Porous biochar-nanoscale zero-valent iron composites: Synthesis , characterization and application for lead ion removal. Sci. Total Environ..

[cit13] Lefevre E., Bossa N., Wiesner M. R., Gunsch C. K. (2016). A review of the environmental implications of *in situ* remediation by nanoscale zero valent iron (nZVI): Behavior , transport and impacts on microbial communities. Sci. Total Environ..

[cit14] Homhoul P., Pengpanich S., Hunsom M. (2011). Treatment of distillery wastewater by the nano-scale zero-valent iron and the supported nano-scale zero-valent iron. Water Environ. Res..

[cit15] Sun Y.-P., Li X., Cao J., Zhang W., Wang H. P. (2006). Characterization of zero-valent iron nanoparticles. Adv. Colloid Interface Sci..

[cit16] Erdem Yayayürük A., Yayayürük O. (2017). Adsorptive performance of nanosized zero-valent iron for V(V) removal from aqueous solutions. J. Chem. Technol. Biotechnol..

[cit17] Wu Y., Yang L., Shi X., Li I., Biazik J. M., Ratinac K. R., Chen D., Thordarson P., Shieh D., Braet F. (2011). The selective growth inhibition of oral cancer by iron core–gold shell nanoparticles through mitochondria-mediated autophagy. Biomaterialsa.

[cit18] Wu Y. N., Wu P. C., Yang L. X., Ratinac K. R., Thordarson P., Jahn K. A., Chen D. H., Bin Shieh D., Braet F. (2013). The anticancer properties of iron core–gold shell nanoparticles in colorectal cancer cells. Int. J. Nanomed..

[cit19] Wu Y. N., Chen D. H., Shi X. Y., Lian C. C., Wang T. Y., Yeh C. S., Ratinac K. R., Thordarson P., Braet F., Bin Shieh D. (2011). Cancer-cell-specific cytotoxicity of non-oxidized iron elements in iron core–gold shell NPs. Nanomedicine.

[cit20] Huang K.-J., Wei Y.-H., Chiu Y.-C., Wu S.-R., Shieh D.-B. (2019). Assessment of zero-valent iron-based nanotherapeutics for ferroptosis induction and resensitization strategy in cancer cells. Biomater. Sci..

[cit21] Dai C., Wang C., Hu R., Lin H., Liu Z., Yu L., Chen Y., Zhang B. (2019). Photonic/magnetic hyperthermia-synergistic nanocatalytic cancer therapy enabled by zero-valence iron nanocatalysts. Biomaterials.

[cit22] Hummers W. S., Offeman R. E. (1958). Preparation of Graphitic Oxide. J. Am. Chem. Soc..

[cit23] Xu Y., Bai H., Lu G., Li C., Shi G. (2008). Flexible graphene films *via* the filtration of water-soluble noncovalent functionalized graphene sheets. J. Am. Chem. Soc..

[cit24] Pu S. , Deng D., Wang K., Wang M., Zhang Y., Shangguan L. and Chu W., Optimizing the removal of nitrate from aqueous solutions *via* reduced graphite oxide – supported nZVI: synthesis , characterization , kinetics , and reduction mechanism, 2019, pp. 3932–3945 10.1007/s11356-018-3813-1 30547335

[cit25] Huang K. J., Wei Y. H., Chiu Y. C., Wu S. R., Bin Shieh D. (2019). Assessment of zero-valent iron-based nanotherapeutics for ferroptosis induction and resensitization strategy in cancer cells. Biomater. Sci..

[cit26] Wei R., Xi W., Wang H., Liu J., Mayr T., Shi L., Sun L. (2017). *In situ* crystal growth of gold nanocrystals on upconversion nanoparticles for synergistic chemo-photothermal therapy. Nanoscale.

[cit27] Cushing B. L., Kolesnichenko V. L., O'Connor C. J. (2004). Recent advances in the liquid-phase syntheses of inorganic nanoparticles. Chem. Rev..

[cit28] Yu J., Hou X., Hu X., Yuan H., Wang J., Chen C. (2019). Efficient degradation of chloramphenicol by zero-valent iron microspheres and new insights in mechanisms. Appl. Catal., B.

[cit29] Phiri M. M., Mulder D., Mason S., Vorster B. (2019). Facile immobilization of glucose oxidase onto gold nanostars with enhanced binding affinity and optimal function. R. Soc. Open Sci..

[cit30] Hui Y., Nan L., Jing-Zhong X., Jun-Jie Z. (2005). A Glucose biosensor based on immobilization of glucose oxidase in chitosan network matrix. Chin. J. Chem..

[cit31] Zhang S., Cao C., Lv X., Dai H., Zhong Z., Liang C., Wang W., Huang W., Song X., Dong X. (2020). A H_2_O_2_ self-sufficient nanoplatform with domino effects for thermal-responsive enhanced chemodynamic therapy. Chem. Sci..

[cit32] Pang Y., Huang Y.-K., Li F., Yang F.-Q., Xia Z.-N. (2016). Rapid screening and evaluation of antioxidants in alkaloid natural products by capillary electrophoresis with chemiluminescence detection. Anal. Methods.

[cit33] Chang C. Y., Hsieh Y. H., Cheng K. Y., Hsieh L. L., Cheng T. C., Yao K. S. (2008). Effect of pH on Fenton process using estimation of hydroxyl radical with salicylic acid as trapping reagent. Water Sci. Technol..

[cit34] Jung Y. S., Lim W. T., Park J. Y., Kim Y. H. (2009). Effect of pH on Fenton and Fenton-like oxidation. Environ. Technol..

[cit35] Zhang W., Deng W., Zhang H., Sun X., Huang T., Wang W., Sun P., Fan Q., Huang W. (2020). Bioorthogonal-targeted 1064 nm excitation theranostic nanoplatform for precise NIR-IIa fluorescence imaging guided efficient NIR-II photothermal therapy. Biomaterials.

[cit36] Hessel C. M., Pattani V. P., Rasch M., Panthani M. G., Koo B., Tunnell J. W., Korgel B. A. (2011). Copper selenide nanocrystals for photothermal therapy. Nano Lett..

[cit37] Jeon B.-C., Nam S.-Y., Kim Y.-K. (2014). Treatment of pharmaceutical wastewaters by hydrogen peroxide and zerovalent iron. Environ. Eng. Res..

[cit38] Rastogi R. P., Singh S. P., Häder D.-P., Sinha R. P. (2010). Detection of reactive oxygen species (ROS) by the oxidant-sensing probe 2′,7′-dichlorodihydrofluorescein diacetate in the cyanobacterium Anabaena variabilis PCC 7937. Biochem. Biophys. Res. Commun..

[cit39] Tang M., Zhu X., Zhang Y., Zhang Z., Zhang Z., Mei Q., Zhang J., Wu M., Liu J., Zhang Y. (2019). Near-infrared excited orthogonal emissive upconversion nanoparticles for imaging-guided on-demand therapy. ACS Nano.

[cit40] Freinbichler W., Bianchi L., Colivicchi M. A., Ballini C., Tipton K. F., Linert W., Corte L. D. (2008). The detection of hydroxyl radicals *in vivo*. J. Inorg. Biochem..

